# Characterizing the developmental transcriptome of the oriental fruit fly, *Bactrocera dorsalis* (Diptera: Tephritidae) through comparative genomic analysis with *Drosophila melanogaster* utilizing modENCODE datasets

**DOI:** 10.1186/1471-2164-15-942

**Published:** 2014-10-28

**Authors:** Scott M Geib, Bernarda Calla, Brian Hall, Shaobin Hou, Nicholas C Manoukis

**Affiliations:** Tropical Crop and Commodity Protection Research Unit, USDA-ARS Daniel K Inouye Pacific Basin Agricultural Research Center, 64 Nowelo Street, Hilo, HI 96720 USA; Department of Plant and Environmental Protection Sciences, University of Hawaii, Manoa, Honolulu, HI USA; Advanced Studies in Genomics, Proteomics and Bioinformatics, University of Hawai’i Manoa, Honolulu, HI 96822 USA

## Abstract

**Background:**

The oriental fruit fly, *Bactrocera dorsalis*, is an important pest of fruit and vegetable crops throughout Asia, and is considered a high risk pest for establishment in the mainland United States. It is a member of the family Tephritidae, which are the most agriculturally important family of flies, and can be considered an out-group to well-studied members of the family Drosophilidae. Despite their importance as pests and their relatedness to *Drosophila*, little information is present on *B. dorsalis* transcripts and proteins. The objective of this paper is to comprehensively characterize the transcripts present throughout the life history of *B. dorsalis* and functionally annotate and analyse these transcripts relative to the presence, expression, and function of orthologous sequences present in *Drosophila melanogaster*.

**Results:**

We present a detailed transcriptome assembly of *B. dorsalis* from egg through adult stages containing 20,666 transcripts across 10,799 unigene components. Utilizing data available through Flybase and the modENCODE project, we compared expression patterns of these transcripts to putative orthologs in *D. melanogaster* in terms of timing, abundance, and function. In addition, temporal expression patterns in *B. dorsalis* were characterized between stages, to establish the constitutive or stage-specific expression patterns of particular transcripts. A fully annotated transcriptome assembly is made available through NCBI, in addition to corresponding expression data.

**Conclusions:**

Through characterizing the transcriptome of *B. dorsalis* through its life history and comparing the transcriptome of *B. dorsalis* to the model organism *D. melanogaster*, a database has been developed that can be used as the foundation to functional genomic research in *Bactrocera* flies and help identify orthologous genes between *B. dorsalis* and *D. melanogaster*. This data provides the foundation for future functional genomic research that will focus on improving our understanding of the physiology and biology of this species at the molecular level. This knowledge can also be applied towards developing improved methods for control, survey, and eradication of this important pest.

**Electronic supplementary material:**

The online version of this article (doi:10.1186/1471-2164-15-942) contains supplementary material, which is available to authorized users.

## Background

The oriental fruit fly (*Bactrocera dorsalis*) is an important agricultural pest in Asia, Africa, and the Pacific, impacting over 150 fruits and vegetables in tropic and sub-tropic regions [1, 2]. In addition, *B. dorsalis* is established across the Hawaiian Islands and is a species of concern in the continental United States, with numerous interceptions and detections made annually, often triggering eradication efforts and quarantines [3–5]. In addition to being an important pest species, *B. dorsalis*, as a member of the family Tephritidae, can serve as an important species for comparison to the well characterized drosophilid group, having diverged from *Drosophila* approximately 70 million years ago [6].

Despite its importance as an agricultural pest, little functional genomic information is available for the species outside of a collection of sequences used for phylogenetic analysis of the genus. The addition of functional genomics information can lead to development of new control strategies, a basic understanding of the biology of the insect, and expansion of the techniques used in research. With the advent of high-throughput sequencing technology, there is increased ability to obtain cost-effective sequence data from non-model organisms, including sequencing from total RNA libraries to target coding genes at particular time points. While collection of this data is fairly straightforward, in order to accurately predict gene models and transcripts, much attention must be given towards assembly and analysis of the data. While this shotgun *de novo* approach to sequencing potentially allows for collection of full length genes, detection of splice variants, and calculation of differential expression between tissues, it can also lead towards assembly of partial gene fragments, erroneous assembly fragments, and mis-assemblies [7–9]. Many RNA-seq experiments report a large number of small transcript fragments, sometimes numbering in the hundreds of thousands, with a small proportion likely to be full length transcripts. This can make it difficult to calculate meaningful expression values, accurately identify transcript isoforms, and limit the utilization of the resulting dataset in downstream functional genomics experiments. For example, for *B. dorsalis* there are several published experiments that have performed *de novo* transcriptome assembly, presented tens of thousands of assembled contigs, but did not publish gene models or transcript sequences as part of their experiment [10–12]. This makes it difficult to utilize the research and limits the application of the research. As an alternative, we attempted to analyze RNA-seq data to produce a high confidence transcript set consisting of full length or near full-length transcripts with strong support for their accuracy. The resulting dataset can be used as a foundation for expanding on the *B. dorsalis* gene set without concerns that the dataset contains erroneous data.

Our approach was to perform comprehensive RNA sequencing on a laboratory colony of *B. dorsalis* with a focus on attempting to capture expression of as many genes and gene splice variants that represent the entire developmental life history. *De novo* transcriptome assembly and analysis of the resulting sequence were performed with a focus on identifying full length gene and splice variants, and then annotating through identification of orthologs in *Drosophila* gene sets. High confidence genes were filtered from potentially erroneously assembled transcripts based on homology to known proteins and read coverage of the transcript. The result produced a high quality reference transcript set for this species for comparative analysis with *D. melanogaster*. This transcriptome can be used as a foundation for functional genomic and population genetic experiments. Further development from other lines of evidence can be used in the future to broaden this gene set.

## Results and discussion

### Sequencing and quality filtering

In total, approximately 101 million paired 100 bp reads were obtained from Illumina GAIIx sequencing, totalling over 20 Gb of data (Table 1). These reads were evenly distributed between all of the libraries sequenced (egg, larvae, pupae, adult male, adult female, mated adult female). All raw reads were submitted to the NCBI Sequence Read Archive under accession numbers SRX261507-SRX261512 associated with BioProject PRJNA167923. After quality filtering and removing low quality reads, approximately 84% of reads remained and were used for assembly and mapping. While filtering provided little improvement to the quality of the beginning of the read, it dramatically increased the quality score of the end of the read from an average q20 score of 24.9 at base 95 in unfiltered reads to average q20 score of 29.2 in filtered reads (Table 1). Filtering also had no dramatic effect on GC content of the data, suggesting there was no bias to the reads that were removed during filtering. *In silico* normalization using the Trinity normalization tool dramatically reduced the number of input reads into the assembly without adversely influencing the kmer abundance from those reads. From ~101,364,649 raw read pairs, filtering and normalization reduced the read abundance to 7,796,491 (~7.7%) reads used as input into the Trinity assembly, greatly reducing the computational requirements for assembly and avoiding complicated de bruin graphs created by low quality sequence or overabundant sequence kmers.

**Table 1:** **Quality filtering of Illumina GAIIx data before assembly and mapping**

				Average quality (interquartile range)												GC content (percent)					
	Number of reads			Before filtering						After filtering						Before filtering			After filtering		
Stage	Before filtering	After filtering	Percent retained	Base 5		Base 50		Base 95		Base 5		Base 50		Base 95		Base 5	Base 50	Base 95	Base 5	Base 50	Base 95
Egg	29899982	24924408	83.4%	36.7	(4)	34.1	(7)	24.9	(25)	37.7	(2)	36.6	(5)	29.3	(8)	40.0	41.1	41.9	38.9	39.8	40.6
Larvae	28913860	23506167	81.3%	36.7	(4)	33.8	(6)	23.5	(33)	37.7	(2)	36.4	(5)	28.2	(8)	41.2	42.8	44.2	40.1	41.4	42.8
Pupae	31954342	26582294	83.2%	36.8	(4)	34.2	(7)	24.6	(33)	37.7	(2)	36.6	(5)	29.0	(8)	40.6	42.0	42.8	39.4	40.6	41.4
Male	41546048	35350086	85.1%	37.4	(2)	35.1	(5)	25.8	(17)	38.2	(2)	37.3	(4)	29.8	(7)	39.7	41.5	42.4	38.5	40.0	41.1
Female	35096592	29240854	83.3%	36.9	(4)	34.4	(7)	24.7	(33)	37.8	(2)	36.7	(5)	29.0	(8)	39.7	41.9	42.8	38.5	40.5	41.4
Mated Female	35318474	29909305	84.7%	36.9	(4)	34.5	(7)	25.3	(18)	37.8	(2)	36.7	(5)	29.4	(8)	38.5	40.9	41.9	37.4	39.6	40.8
Total	202729298	169513114	83.6%	36.9	(3.5)	34.4	(6.5)	24.9	(26)	37.8	(2)	36.7	(4.8)	29.2	(7.8)	39.9	41.7	42.6	38.7	40.3	41.3

### *De novo*transcriptome assembly and transcript filtering

**Figure 1:**
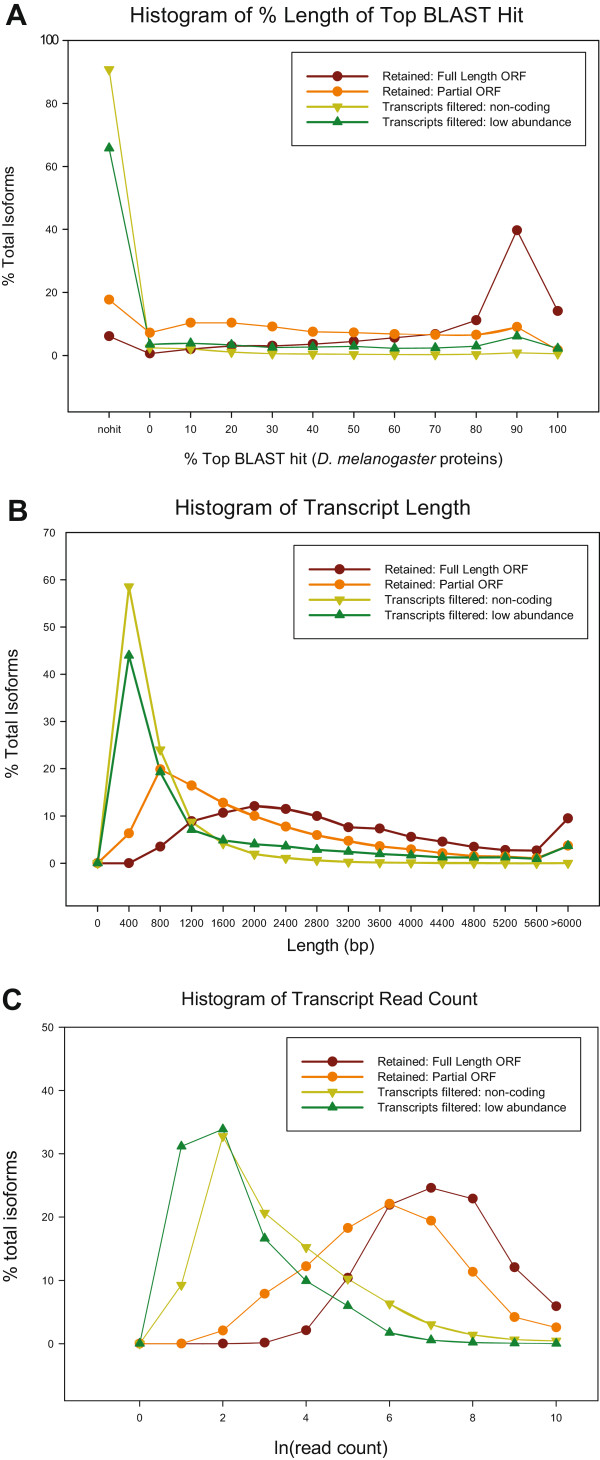
**Comparative evidence for filtering of** ***de novo*** **assembly.** Lines represent retained unigenes (either containing a full or partial ORF) or transcripts discarded based off of lack of evidence of coding region or due to low expression values. **A)** Proportion length of *B. dorsalis* putative transcripts relative to highest scoring blast alignment to *D. melanogaster* proteins. **B)** Length distribution of transcripts. **C)** Relative log read count expression values of transcripts.

The *B. dorsalis* transcriptome was reconstructed using the Trinity assembly pipeline with all filtered reads from all libraries pooled into one dataset [9, 13]. This raw assembly yielded 80,346 contigs, with an N50 contig size of 2,802 bases, 31,321 contigs greater than 1000 bp, and a transcript sum of 109.4 Mb. While not all assembled contigs produced by Trinity represent true transcripts in *B. dorsalis*, this contig set was used as a starting point for defining the transcriptome present in our sample. Filtering based off of read abundance and component isoform percentage removed 35,729 sequences, leaving 44,617 remaining. Further filtering through identification of likely coding sequence based on ORF prediction identified 6,864 genes containing a complete ORF (unique genes defined as sequences with unique comp##_c# identifier from Trinity (http://trinityrnaseq.sourceforge.net/)) with 13,017 isoforms giving complete ORFs. In addition, 3,935 genes only contained a partial ORF (missing either 5' or 3' end or both) with 7,649 isoforms identified in those genes, giving a total of 20,666 transcripts across 10,799 genes, with an N50 transcript size of 3,460 bp and transcript sum of 62.08 Mb. While Trinity assembled an additional 56,785 contigs, these were discarded by the above filtering because they lacked a likely coding sequence or did not have significant read coverage. While in some cases these may contain partial gene fragments, we chose to exclude them from further analysis as the majority consist of short, low read count data with low blast homology to known proteins in *D. melanogaster* (Figure 1A-C). In contrast, the majority of the retained transcripts had a significant number of reads mapping to them, with full length transcripts being longer, and having a higher alignment percentage to known *Drosophila* proteins when compared to the partial transcripts (Figure 1A-C).

### Read abundance based expression analysis across developmental stages

**Figure 2:**
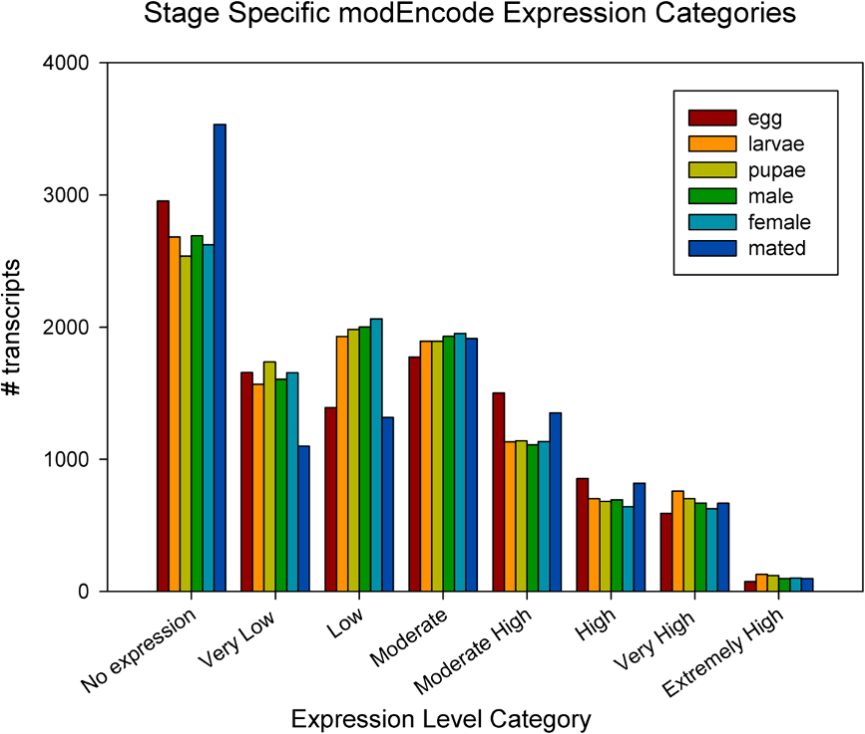
**Distribution of expression categories across** ***B. dorsalis*** **developmental stages.** Placement of unigenes across modENCODE expression categories, separated by developmental stage.

Stage specific expression values were calculated for each developmental library as TMM normalized FPKM values. Unique genes were classified with the modENCODE FPKM expression categories (very low, low, moderate, moderate high, high, very high, extremely high) utilized by flybase.org with the same FPKM expression level calculations and category bins used in *Drosophila* [14, 15]. Distribution of expression categories across developmental stages are presented in Figure 2. From this, genes were categorized as constitutively expressed in all libraries, stage-specific or expressed across several stages. Constitutive highly expressed genes were defined as those genes having an expression level of "moderately high" or higher in all 6 samples. Under this definition, we identified 1,347 constitutive highly expressed genes in our dataset. Stage specific genes were defined as those that are "moderately expressed" or higher in one library and “low” or "very low" in the other 5 libraries. A total of 310 unigenes were declared stage specific. Finally, genes expressed across two, three, four, or five libraries were also calculated, as many genes may be expressed during several related libraries that could constitute a biologically relevant developmental stage. This identified 395 genes expressed at least moderately high in two libraries compared to very low in the rest of the libraries, 186 genes with this same pattern in three libraries, 156 in 4 libraries and 136 in 5 libraries. The majority of the genes in the combined transcriptome dataset were found constitutively expressed at a low level (“low” or “very low”, 8,269 genes). A breakdown of the number of stage specific genes is listed in Table 2, and a matrix of stage specific and constitutively expressed genes is provided in Additional file 1.

**Table 2:** **Number of unigenes present in expression level categories**

Stage	Number of unigenes	Percentage of total unigenes (%)
**All stages:**		
Constitutive highly expressed genes	1,347	12.47
Constitutive low	8,269	76.56
**Single Stages:**		
Egg specific	29	0.26
Larvae specific	136	1.25
Pupae specific	110	1.01
Mated Female specific	35	0.324
Adults (Female and male) specific	50	0.46

Lists of genes present in each category is provided as matrices in Additional file 1. Constitutive highly expressed genes have modENCODE expression values of “High” or greater across all stage specific samples. Sparsely expressed genes have a value of “Low” or lower.

**Figure 3:**
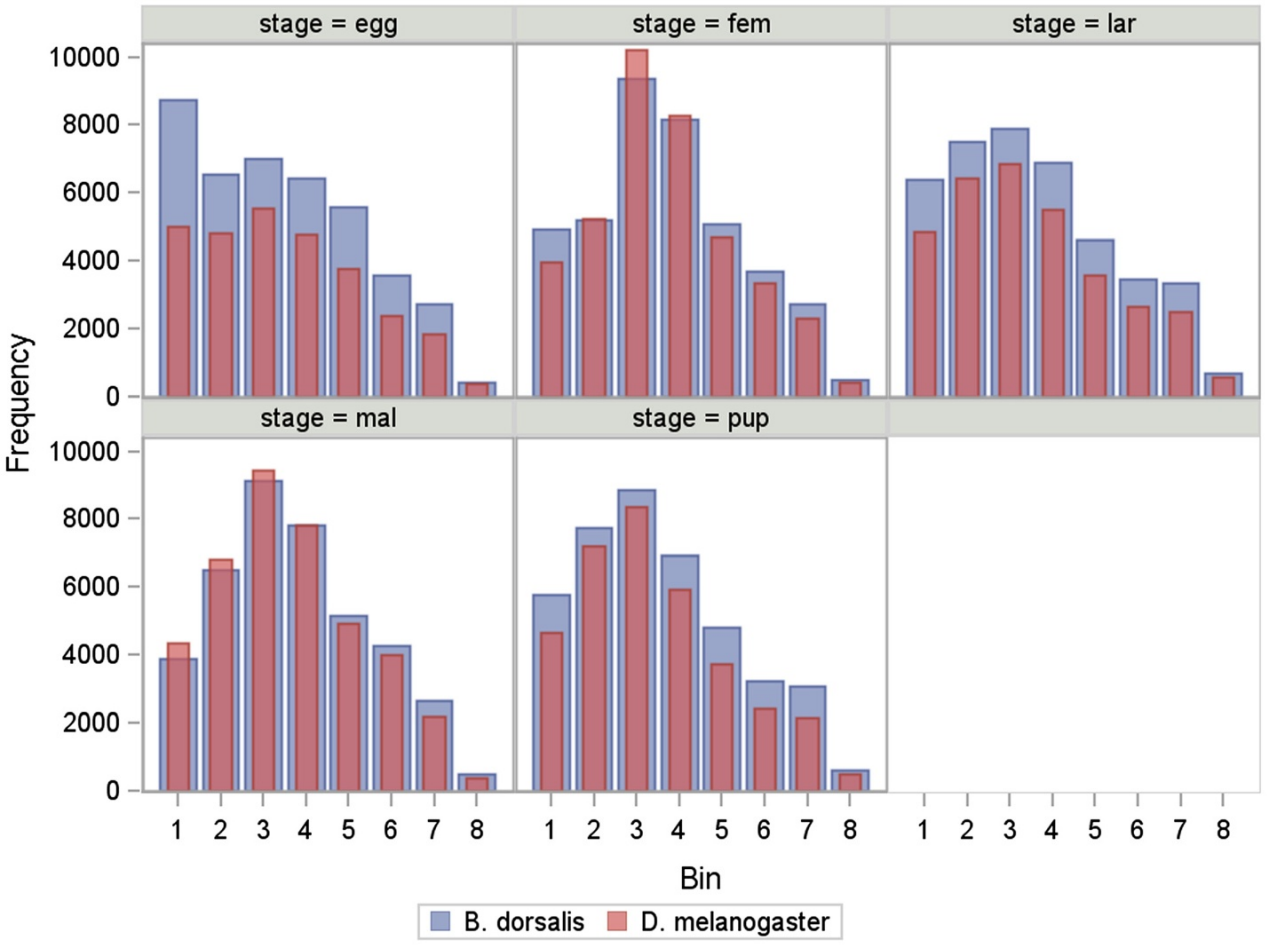
**Comparison of expression level distribution between** ***B. dorsalis*** **and** ***D. melanogaster*** **utilizing modENCODE expression categories.** Numbers below bars represent modENCODE expression categories as follows: 1- No Expression, 2- Very Low, 3- Low, 4- Moderate, 5- Moderate High, 6- High, 7- Very High, 8- Extremely High. Frequency of all *B. dorsalis* unigene models were compared to all gene level expression values derived from the *D. melanogaster* modENCODE project.

Using the same classification described above, the number of genes falling into each of the expression level categories for each stage was compared with that of *D. melanogaster* (Figure 3), demonstrating strong similarity of distribution of gene expression levels in both species. While this result helps to corroborate the expression values presented for *B. dorsalis*, it additionally highlights the intra-species conservancy of expression levels for the majority of transcripts, suggesting stage-specific functional conservation between the identified genes in *B. dorsalis* and *D. melanogaster* (Figure 3).

**Figure 4:**
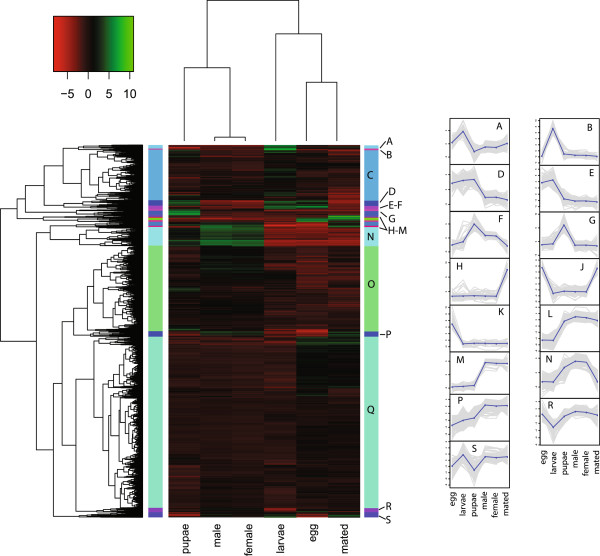
**Two dimensional cluster of expression profiles of unigenes in** ***B. dorsalis*** **across developmental stages.** Two dimensional clustering of developmental stages by expression patterns of the 10,799 unigenes identified. Vertical cluster of unigenes sub-clustered into unigenes of similar expression profile, subclusters color coded on vertical bar adjacent to heatmap, and cluster expression profiles presented as inset figures **A-S**. Vertical axis of inset figures is log2(median centered FPKM values).

Another complementary approach to compare expression between stages was employed through hierarchical clustering of the gene expression values between libraries, and then defining subclusters that share similar expression patterns across libraries. From the hierarchical clusters, 19 subclusters were created by splitting the tree at clusters that shared 45% of the tree height. These subclusters were visualized by plotting the median centered FPKM values (Figure 4). In this case, expression patterns are not focused specifically on the most highly expressed genes as in the first approach, but rather on genes that exhibit a change in expression based on median-centered values, regardless of the level of expression. Some of the defined clusters did not clearly describe a stage specific expression pattern such that no expression values were distributed significantly outside of the median (e.g. cluster Q, Figure 4); others, however, showed clear differences in expression between libraries. These patterns are highlighted as inset figures in Figure 4, and a list of genes present in each cluster is provided as supplementary data (Additional file 2).

### Putative unigenes orthologous to *D. melanogaster*genes

**Figure 5:**
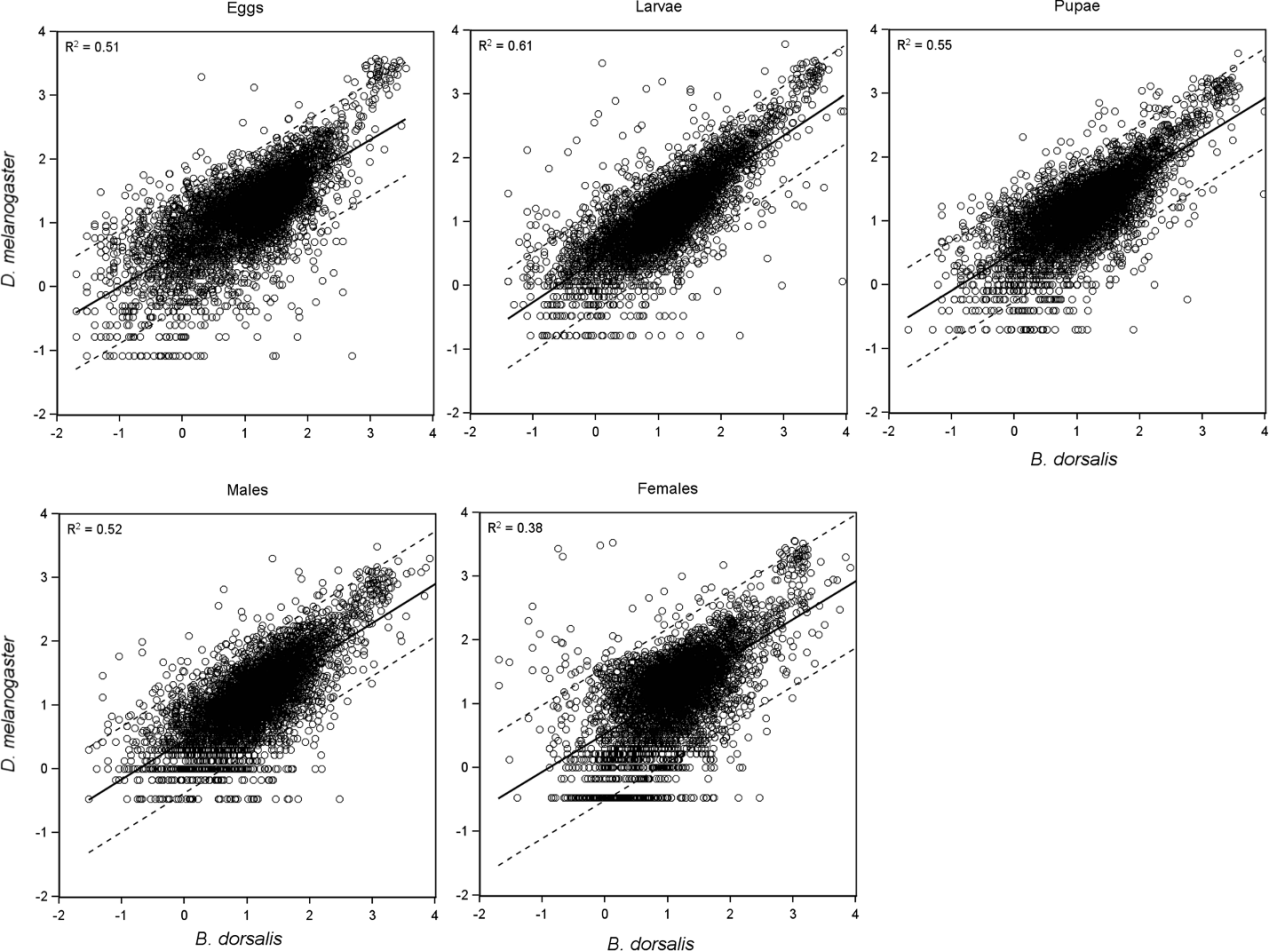
**Regression analysis of log expression values between orthologous unigenes in** ***B. dorsalis*** **and** ***D. melanogaster*** **across developmental stages.** Linear regression represented as solid line with 95% confidence interval indicated with dashed lines. Each *B. dorsalis* unigene with putative ortholog identified in *D. melanogaster* developmental stage was plotted against the putative ortholog based off of log(10) expression values. Detailed list of each unigene outside of the confidence interval is presented in Additional file 3.

A set of 5,681 putative orthologous genes were identified in our *B. dorsalis* dataset through reciprocal BLAST alignment with the *D. melanogaster* protein set. We anticipate this number to be an underestimation of actual ortholog representation, as utilizing an RNA-seq assembly increases the chance for multiple unigenes. Expression levels in terms of normalized FPKM values were compared between the putative orthologs by plotting the data along a regression curve. A strong linear relationship was observed between the expression values of orthologs (Figure 5). To identify orthologous proteins with potential difference in expression between the two species, a 95% interval was calculated along the regression. A conservative approach was taken to avoid the identification of false orthologs, and at times this approach may not identify true orthologs. This is particularly true when using *de novo* assembled RNA-seq data, which commonly has a large proportion of extraneous or superfluous transcripts assembled due to genetic heterogeneity of the sample, sequence error, and/or relaxed transcript reconstruction parameters of the software. By filtering the assembly to the approximate expected unigene and transcript density, over 5,600 putative orthologs were identified. Through linear regression analysis between expression values of the orthologs in *B. dorsalis* and *D. melanogaster*, we found a strong linear correlation at each developmental stage. Of the unigenes falling outside of the confidence interval of the regression, a significant proportion were identified as constitutive low expression. This bias seems largely due to a large number of unigenes with higher expression (although still constitutively low) in *B. dorsalis* compared to *D. melanogaster* and are likely not biologically relevant. In contrast, there are 4,234 unigenes (~75%) that are always within the confidence interval and about 25% that are conversely outside in at least one developmental stage (Additional file 3). Only 30 unigenes were consistently outside of the confidence interval in all 5 developmental stages. This strong homology in expression patterns between *B. dorsalis* and *D. melanogaster* for the putative orthologs suggests conserved functions between species. Of course, this is biased, as additional unigenes that may be orthologs were not identified by our analysis, and it is possible that those may be enriched for proteins that have modified function or expression patterns, and may not have passed the threshold set. Despite some genes not falling within the confidence interval of the regression, Pearson correlation values (rho) at a p < 0.001 were calculated and both data sets were found to have a strong correlation. Both the regression and the correlation support assembly accuracy and relative completeness. For genes outside of the confidence, while considered potential orthologs, may have different functions or temporal expression patterns between the two species, or may be more sensitive to external variables that potentially differed between rearing conditions.

### Functional annotation

A large proportion of the transcripts assembled and retained after filtering were able to be functionally annotated through BLAST homology to proteins in the UniProt Swiss-Prot database and the *D. melanogaster* protein set or HMM profiles in Pfam-A. Further, when possible, gene ontology, KOG, and COG terms were added. In total, 17,093 transcripts (72.5% of total) were annotated from BLAST homology to Swiss-Prot with a qualified gene name, whereas 20,713 transcripts (87.9% of total) had significant alignment to *D. melanogaster* proteins (r5.44), encompassing 10,686 proteins from 9,004 unique gene models in *D. melanogaster*, and representing 65% of known *D. melanogaster* genes. In contrast, 16,612 transcripts were annotated with Pfam domains, encompassing 3,846 unique Pfam identities.

### Identification of transcription factors in the *B dorsalis*genome

Spatial and temporal patterns of gene expression are in part regulated by transcription factors. Transcription factors (TFs) were searched in our dataset initially by using the Gene Ontology annotations and further curated using Pfam. Genes with GO terms corresponding to: GO:0003700 (sequence-specific DNA binding transcription factor activity), GO:0006355 (regulation of transcription, DNA-templated), and GO:0003705 (RNA polymerase II distal enhancer, sequence-specific DNA binding transcription factor activity) were selected and Pfam annotations were used to curate and classify the selected list of genes. A total of 1,265 transcripts were classified as TFs, roughly 6% of the transcriptome. This percentage is comparable with the number of TFs estimated in *D. melanogaster* [16]. The 1,265 identified sequences corresponded to one of 305 unique domains. The most abundant TFs corresponded to Zinc finger domains, followed by homeobox domains and helix-loop-helix proteins (Table 3). It is worth noting that several sequences may be false positives. For the purposes presented here, transcription factors were defined as DNA binding domains, however several proteins exist that possess motifs similar to DNA binding factors and yet may be unrelated. Similarly, several true transcription factors might be missing from the analysis.

**Table 3:** **Classification of transcripts annotated as transcription factors**

Transcription factor domain class	Number of transcripts	Relative percentage of total TF trascripts
Zinc finger	330	26.1
Homeobox	64	5.1
Helix-loop-helix	55	4.4
BTB/POZ	44	3.5
Bzip	39	3.1
Myb	24	1.9
WD40	19	1.5
Basal TFs	18	1.4
Fork-head	18	1.4
Bromodomain	15	1.2
Helicase	13	1.0
other	625	49.4
Total	1264	100.0

Distribution of transcription factors. The eleven most abundant domain classes are listed, but 305 unique domains in total were identified. Transcripts belonging to classes not specifically listed were identified by fewer than 13 unique transcripts and pooled into the “other” category.

### Detoxification genes in *B. dorsalis*transcriptome

An important aspect of insect evolutionary adaptation is their ability to remove acquired toxic compounds and protect the organism. To identify genes related to detoxification in *B. dorsalis*, genes linked with Phases I and II in mammalian detoxification pathways were mined from the transcriptome dataset through sequence homology. Cytochrome P450 monooxygenases (P450s) accounted for 163 transcripts in 93 unique unigenes, approximately equating the 90 genes found in Drosophila from this superfamily [17]. These 93 unigenes belong to 12 families (Table 4). While many P450 proteins are known to be involved in detoxification, the superfamily consists of a wide array of enzymes with distinct functions [17, 18]. This is not the first report of characterization of P450’s in *B. dorsalis*, with previous research reporting as many as 90 unigenes to as few as 51 from shotgun transcriptome experiments [10, 12, 19]. Inconsistent database deposition of sequences obtained in previous reports makes it difficult to make comparisons and draw conclusions across the respective studies. In one case, multiple P450s were verified through cloning [20], and 60 P450’s were reported from the olive fly *B. oleae* [21]. The overall consensus suggests strong consistency in the diversity and abundance of P450’s in *B. dorsalis* and *D. melanogaster*.

**Table 4:** **Distribution of detoxification annotations in** ***B. dorsalis*** **transcriptome including cytochrome P450, glutathione S-transferases, and UDP-glucuronosyltransferases**

Gene class		Family	Number found in ***B. dorsalis*** ^***α***^
P450s	P450 monooxygenases	CYPIV	21
		CYPIII	1
		CYPIX	2
		CYPVI	29
		CYPXII	11
		CYPXLIX	1
		CYPXVIII	1
		CYPXXVIII	2
		CYPCCCI	1
		CYPCCCIII	1
		CYPCCCIV	2
		CYPCCCIX	4
		CYPCCCV	1
		CYPCCCVI	1
		CYPCCCVIII	1
		CYPCCCX	1
		CYPCCCXI	1
		CYPCCCXIII	6
		CYPCCCXIV	1
		CYPCCCXV	1
		CYPCCCXVII	1
		CYPCCCXVIII	2
GSTs	glutathione S-transferases		34^β^
UGTs	UDP-glucuronosyltransferases		23

^***α***^Breakdown of individual unigenes annotated to each class available in Additional file 4.

^β^For GSTs, 4 contained only N-terminal domains, 4 only C-terminal domains and 26 genes encompassed both domains.

### Glutathione-S-transferases (GSTs) and UDP-glucuronosyltransferases

(UGTs) constitute phase II of the mammal detoxification pathway, usually by mitigating the effects of the oxygen radicals produced by phase I [22]. Our transcriptome profiling detected 34 unique GSTs domains, 26 of which had both N- and C- terminal domains and eight with homology to either the C-terminal region only or the N-terminal region (four and four respectively). This is comparable to a previous report that identified 37 unigenes with GST domains [12]. Additionally, 23 UGTs were found (Table 4). Furthermore, data from a *D. melanogaster* microarray experiment designed to profile genes in the CncC/Keap1 pathway was obtained [23]. The CnC/Keap pathway is presumably the central pathway for detoxification in *D. melanogaster* and has little evolutionary relationship with detoxification in mammals. The data was cross-referenced with our identified orthologous genes in *B. dorsalis*. Among the genes that changed in expression in response to CncC/Keap pathway activation in *D. melanogaster*, 141 appear to be present in *B. dorsalis* with most constitutively expressed (either at a high or low degree) across all stages, with no specificity to their expression pattern (Additional file 4).

### Sex determination transcripts

Genes involved in sex-determination can be used to develop pest control tools as well as to improve the existing sterile insect technique (SIT) [24]. To identify sex-determinant genes, amino acid sequences of 67 transcripts corresponding to 14 genes known to be involved in sex determination in *D. melanogaster* [25, 26] were used to search our *B. dorsalis* transcriptome dataset trough BLASTp (e-value cutoff 1E-10). This resulted in 169 *B. dorsalis* unique transcripts with high sequence similarity to at least one of the *D. melanogaster* peptide sequences (Additional file 5).

The genes *Sex lethal* (*Sxl*) and *Notch* (*N*) had the highest number of transcripts with high sequence similarity in *B. dorsalis*. The gene *transformer* (*tra*) was not found in our transcriptome assembly, and only two transcripts matched *transformer 2* (*tra2*) (which has 7 transcripts in *D. melanogaster*). The gene *tra2* was found to be critical for sex-determiantion in *Anastrepha* fruit flies [27] but needs to interact with *tra*. While *tra* was found to be present in another *Bactrocera* species, *B. oleae* [28], it may not have been actively expressed in the samples we tested, despite our effort to include as many developmental stages as possible. A previous report investigated reproduction and development in *B. dorsalis* utilizing RNA-seq, but did not investigate sex-determination [11]. Additionally, putative sex determinant *D. melanogaster* orthologs and transcripts with sequence similarity to other species were mined from the annotation output and the *D. melanogaster* orthologous datasets. The total number of genes, transcripts and orthologs found are in Additional file 5.

## Conclusions

The sequencing of stage-specific messenger RNA from the fruit fly *B. dorsalis* allowed for the construction of a transcriptome that when compared with *D. melanogaster* allowed for the identification of 20,666 transcripts across 10,799 unigenes. This is comparable to the current genome annotation derived *D. melanogaster* transcript set, which includes 15,504 genes, 25,205 isoforms, N50 isoform length of 3,633 and an isoform sum of 68.46 Mb. Utilizing binning expression nomenclature utilized by the modENCODE project, 65% of the unigenes identified were expressed at a constitutively low level (Figure 2). In addition, the relative distribution of unigenes across bins throughout the developmental life history in *B. dorsalis* is very similar in pattern to the distribution seen in *D. melanogaster* across the same developmental stages. Despite this, distribution does not directly demonstrate similarity between species at a gene or unigene level, so putative orthologs were identified using a reciprocal blast approach.

Functional annotation of the transcriptome yielded a large diversity of functions. The focus of this study was on proteins related to DNA binding, detoxification, and sex determination. This analysis demonstrates the utility of a *D. melanogaster* based annotation approach for closely related species, and potentially that functions or processes in *Drosophila* will largely translate to Tephritide flies. Despite this, direct transfer of *Drosophila* annotations is not always sufficient for inferring function. For example, genes involved in sex-determination are potential targets for the development of novel control strategies and the improvement of the sterile insect technique (SIT) in Tephritids. Orthology to *Drosophila* should be used with caution, as sex determination is highly diverged among dipeteran insects. For example, *Sxl* in tephritids is not regulated in a sex-specific fashion; rather the *Sxl* transcript is present in both sexes. To address this point, further studies utilizing more accurate and sensitive techniques will be needed to validate and verify the expression patterns of genes of interest throughout the life history of *B. dorsalis*. In addition, functional genomic approaches will lead to a better understanding of the similarities and differences between Tephritids and Drosophilids and serve as a foundation for developing new control techniques for pestiferous true fruit flies.

## Methods

### *Bactrocera dorsalis*colony rearing and stage collections

*Bactrocera dorsalis* eggs used in this experiment were obtained from the USDA-ARS-Pacific Basin Agricultural Research Center research colony "Punador" (Hilo, HI, USA) maintained on conventional mill feed diet [29]. This colony is derived from wild flies collected in Puna, Hawaii in 1984 and has since been maintained in the laboratory on artificial diet. These eggs were transferred to liquid based artificial diet [30, 31] and insects reared to obtain a collection of samples representing the entire life cycle of this insect. Typically, when reared on artificial diet in colony, eggs take approximately 1–3 days to hatch; insects stay in the larval stage 8–10 days, are in pupal stage for 10 days, and survive as adults for several weeks. Maturation of adults takes approximately 7 days, and adults begin mating after that time. Samples were collected in the following manner in order to produce RNA samples that are representative of an entire life stage, rather than just a single point in time during development.

Fresh eggs from colony flies were maintained at room temperature for 3 days until complete hatch. During that time, a daily sample of eggs was taken. The samples were snap frozen in liquid nitrogen and then stored at -80°C. After hatch, 12 separate containers of liquid artificial diet were set up with larvae, maintained at room temperature, and each day for 10 days one container was used to collect larvae. Liquid diet containing larvae was strained through a mesh sieve and larvae were quickly rinsed in distilled water. Larvae were then collected in microfuge tubes and snap frozen in liquid nitrogen and stored at -80°C. The larvae in the remaining two containers were allowed to pupate following standard methods. Pupae were collected and daily pupal samples were collected for 10 days; each day pupae were snap frozen and stored. Adult cages with sugar water and torula yeast were then set up with mature pupae, and adults were allowed to emerge following standard protocols [32]. At this time, adults were sexed and males and females separated. Daily collections of virgin males and females were made for 7 days and samples immediately snap frozen. After that time, males and females were combined into a single cage for mating. Mating cages were carefully observed and mated females were identified, collected, and frozen.

### RNA extraction and sequencing

Total RNA was extracted from each collection day spanning from egg to adult using the Qiagen RNeasy Plus Mini Kit (Qiagen Inc., Valencia, CA) following the manufacturer’s procedures with the following modifications. Approximately 30 – 50 mg of liquid nitrogen snap-frozen tissue was placed in 600 μl Buffer RLT with 1% β-mercaptoethanol and ground carefully with disposable micropestel in a microfuge tube. This solution was then passed through a QIAshredder column and then through a gDNA Eliminator column. In addition, before final elution, on column RNase-Free DNase treatment was performed to ensure full removal of genomic DNA from sample. RNA concentration and quality was assessed using a Qubit fluorometer (Invitrogen Corp., Carlsbad, CA, USA) as well as an Agilent 2100 Bioanalyzer (Santa Clara, CA, USA) following standard protocols. From these RNA extractions, stage-specific samples were created by pooling intra-stage samples at an equal concentration ratio. In total, six stage-representative samples were collected representing egg, larval, pupal, adult male, adult female, and mated adult female stages. Each of these total RNA samples was prepared for sequencing using the TruSeq RNA Sample Preparation Kit (Illumina Inc., San Diego, CA, USA) and sequenced on an Illumina GAIIx to produce approximately 14 – 20 million 2 × 101 bp PE reads per sample library.

### Raw read quality filiter, *in silico*library normalization and *de novo*transcriptome assembly

Pre-filtering the reads for quality is critical to obtaining a high quality assembly and produce accurate RNA-Seq expression data. Reads were filtered that contained a Phred score below 20 across more than 20% of the bases using the fastx-toolkit fastq_quality_filter script (http://hannonlab.cshl.edu/fastx_toolkit/commandline.html). These quality-filtered reads were then normalized to reduce redundant read data and discard read errors using Trinity's normalize_by_kmer_coverage.pl script with a kmer size of 25 and maximum read coverage of 30. The resulting normalized reads were used to create a *de novo* transcriptome assembly using the Trinity *de novo* transcriptome assembly pipeline (r2012-10-05) [19, 33]. The Trinity pipeline (Inchworm, Chrysalis, and Butterfly) was executed using default parameters, implementing the --REDUCE flag in Butterfly and utilizing the Jellyfish k-mer counting approach [33]. Assembly completed in 3 hours and 13 minutes on a compute node consisting of 32 Xeon 3.1 GHz cpus and 256 Gb of RAM available to the software on the USDA-ARS Pacific Basin Agricultural Research Center Moana computer cluster (http://moana.dnsalias.org).

### Assembly filtering and gene prediction

The output of the Trinity pipeline is a fasta formatted file containing sequences defined as a set of transcripts, including alternatively spliced isoforms determined during graph reconstruction in the Butterfly step. These transcripts are grouped into gene components, which represent multiple isoforms across a single unigene model. While many full-length transcripts are expected to be present, it is likely that the assembly also consists of erroneous contigs, partial transcript fragments, and non-coding RNA molecules. This collection of sequences was thus filtered to identify contigs containing full or near full-length transcripts or likely coding regions and isoforms that are representative at a minimum level based off of read abundance. Pooled non-normalized reads were aligned to the unfiltered Trinity.fasta transcript file using bowtie 0.12.7 through the alignReads.pl script distributed with Trinity. Abundance of each transcript was calculated using RSEM 1.2.0 utilizing the Trinity wrapper run_RSEM.pl [13, 34]. Through this wrapper, RSEM read abundance values were calculated on a per-isoform and per-gene basis. In addition, the percent composition of each transcript component for each gene is calculated. From these results, the original assembly file produced by Trinity was filtered to remove transcripts that represent less than 1% of the RSEM based expression level of its parent gene or transcripts with TPM (Transcripts per Million) value below 0.5. This filter set discarded is referred to as "transcripts filtered: low abundance" in figures and tables and the retained transcripts used as input for further filtering.

Coding sequence was predicted from the filtered transcripts using the transcripts_to_best_scoring_ORFs.pl script distributed with the Trinity software from both strands of the transcripts. This approach uses the software Transdecoder (http://transdecoder.sourceforge.net/) which first identifies the longest open reading frame (ORF) for each transcript and then the 500 longest ORFs are used to build a Markov model against a randomization of these ORFs to distinguish between coding and non-coding regions. This model is then used to score the likelihood of the longest ORFs in all of the transcripts, reporting only those putative ORFs which outscore the other reading frames [13]. Thus, the low abundance filtered transcript assembly was split into contigs that contain complete transcripts (Retained: Full Length ORF), contigs containing transcript fragments with predicted partial ORF (Retained: Partial ORF) and contigs containing no ORF prediction (Transcripts filtered: non-coding). The resulting retained transcript sets (partial and full) were merged and subjected to annotation and utilized in subsequent analysis. In addition, sequence length and read count histograms were created for each of the four filter categories described above to help visualize the effect of filtering on retention of transcripts (Figure 1A-B).

### Gene annotation

The filtered transcripts were annotated using the UniProtKB/Swiss-Prot database, Pfam-A, eggNOG, and gene ontology utilizing the Trinotate annotation pipeline. In addition, homologous proteins in *Drosophila melanogaster* were identified. The filtered transcript set was first subjected to blastp search against the UniProtKB/Swiss-Prot database using blast-2-2-26+ with an e-value cutoff of 1.0E-5. In addition, protein domains were identified through searching the Pfam_A database using HMMER 3.0. Signal peptides and transmembrane domains were annotated with SignalP 4.1 and TMHMM 2.0 respectively. The resulting outputs were loaded into a Trinotate database, where eggNOG and Gene Ontology terms were annotated and the resulting annotation set was exported as a delimited file for further analysis (Additional file 6). In addition, transcripts were subjected to BLASTx search against the current *D. melanogaster* protein set (Flybase.org, Dmel-r5.44) and uniref90 using an e-value cutoff of 1.0E-5 to identify homologous genes [35]. In addition, percent alignment length of the query transcript against the top *D. melanogaster* alignment was calculated and binned for each filtering category and plotted (Figure 1C). To further define putative orthologs to *D. melanogaster* proteins, reciprocal BLASTp alignment was performed between protein sequences in the *B. dorsalis* transcriptome assembly and all known *D. melanogaster* proteins. Putative orthologs were defined only when reciprocal top scoring hit (at unigene/gene level) between both searches were the same and were unique. This yielded 5,681 unigenes orthologous with *D. melanogaster* genes. An additional 4,186 unigenes were members of a reciprocal hit that was not unique, with multiple unigene/genes having equal score. These were not included as putative orthologs, as they may represent paralogous proteins or other situations without a 1:1 relationship, and thus this is a conservative, yet largely accurate analysis [36]. Putative orthologs are presented in Additional file 7, as a cross reference between flybase gene ID and *B. dorsalis* unigene identifier. The resulting annotated transcriptome was converted to Genbank .tbl format using a beta-release of the Genome Annotation Generator (http://genomeannotation.github.io/GAG/) and transvestigator (http://genomeannotation.github.io/transvestigator/) and submitted to NCBI under Transcriptome Shotgun Assembly (TSA) GAKP00000000 associated with Bioproject 167923.

### Read library mapping and expression analysis

Because the Trinity assembler is able to accurately predict splice isoforms, gene and isoform expression quantification was performed using the RSEM (RNA-Seq by Expectation Maximization) software package (v1.1.15) which is particularly well suited to work with multiple isoforms where the same read may map to multiple sequences [34]. The filtered transcript set from Trinity was used for analysis that only contained contigs containing likely coding sequence (full and partial ORFs) to avoid skewing expression quantification results with non-coding and fragmented data. Quality filtered reads from each sequencing library (egg, larvae, pupae, adult male, adult female, mated adult female) were independently mapped to the reference transcriptome assembly created by Trinity using bowtie (v 0.12.7) [37] using the alignReads.pl script distributed with Trinity. The resulting bam formatted mapping files were sorted and used to produce fragment abundance estimation by RSEM [34]. Transcript abundance values were produced as expected read count, and they were normalized using trimmed mean of M values (TMM) methods and transformed into fragments per feature kilobase per million reads mapped (FPKM) for each gene and the individual isoforms that compose each gene for each developmental library using scripts provided by Trinity [38, 39]. These TMM normalized FPKM values were appended to the annotation information, and binned into expression level categories based off of FPKM values (No Expression, very low, low, moderate, moderately high, high, very high, and extremely high) following the approach and nomenclature used to describe modENCODE expression data in FlyBase2012_06 (David Emmert and William Gelbart, personal communication) (Figure 4, Additional file 1) [14, 35, 40]. TMM normalized FPKM values were also used to create a dissimilarity matrix based on Euclidean distance utilizing the dist function in R and this matrix was used to perform cluster analysis using the complete agglomeration method in the hclust R function. These clusters and the TMM normalized FPKM values were used to create a heatmap of expression levels sorted by both transcript and sample clusters utilizing the R enhanced heatmap (heatmap.2) function. Each cluster in the expression heatmap describes a different developmental expression pattern shared by all genes present in that cluster. Both TMM normalized and read count matrices are available under the NCBI Gene Expression Omnibus (GEO) under accession GSE46310 associated with BioProject PRJNA198716.

## Electronic supplementary material

Additional file 1: **Expression categories across developmental stages for unigenes.** An .XLSX file based off of modENCODE expression categories, listing all unigenes and if they are constitutively expressed, or expressed only during certain developmental stages. (XLSX 589 KB)

Additional file 2: **Unigene expression matrix of major clusters in Figure** 4 **.** An .XLSX file containing individual tabs for each cluster present in Figure 4 (clusters A-S). Each cluster specific matrix contains the unigene identifiers present in the cluster and the log2(median centered FPKM values) across the 6 developmental stages. (XLSX 1019 KB)

Additional file 3: **List of unigenes outside of confidence interval in regression analysis between expression levels of putative orthologs between** ***B. dorsalis*** **and** ***D. melanogaster.*** (XLSX 222 KB)

Additional file 4: **Unigenes associated with detoxification.** An .XLSX file containing unigenes annotated as relating to detoxification in *B. dorsalis.* (XLSX 70 KB)

Additional file 5: **Transcripts associated with sex determination identified in** ***B. dorsalis.*** An .XLSX file containing results from comparative analysis of sex determination genes in *B. dorsalis* utilizing putative orthologs identified in *D. melanogaster*. (XLSX 11 KB)

Additional file 6: **Functional annotation table of peptide sequences from predicted open reading frame of transcripts.** Tab delimited text file containing annotations associated with peptide sequences of each transcript associated with unigene components. Includes top blast hit against UniProtKB/SwissProt, Pfam domains, results from SignalP and TmHMM analysis and COG and GO terms associated with the sequence. Functional annotations were also submitted with the NCBI TSA submission. (ZIP 7 MB)

Additional file 7: **Putative orthologous genes/unigenes between** ***D. melanogaster*** **and** ***B. dorsalis.*** An .XLSX file containing *B. dorsalis* unigene identifier and *D. melanogaster* Flybase gene ID (FBgn#######). First tab presents putative orthologs as defined as reciprocal best unique blast hit, and second tab presents unigenes with top scoring hit, but not considered orthologous due to multiple top scoring unigenes/genes (non-unique hit). (XLSX 218 KB)
